# The Use of Pictorial Recall Aids Modifies Dietary Assessment Results: Experiences from Quantitative 24-hour Dietary Recalls of Young Children in Nepal and Senegal^☆^

**DOI:** 10.1016/j.cdnut.2024.104452

**Published:** 2024-08-30

**Authors:** Anna Vanderkooy, Elaine L Ferguson, Nisha Sharma, Ndèye Yaga Sy, Rosenette Kane, Alissa M Pries

**Affiliations:** 1 Helen Keller International, Dakar, Senegal; 2 Department of Population Health, London School of Hygiene and Tropical Medicine, London, United Kingdom; 3 Helen Keller International, Kathmandu, Nepal; 4 Helen Keller International, NY, United States

**Keywords:** recall aid, memory aid, recall bias, memory error, dietary assessment, 24-h recall, children, measurement, Nepal, Senegal

## Abstract

**Background:**

Recall bias or memory error is a known limitation of recall-based dietary assessment methods and can lead to substantial misrepresentation of diets. Pictorial recall aids have been used by various studies to mitigate recall bias, but their effect on quantitative, 4-pass 24-h dietary recall (24HR) results has not yet been measured.

**Objectives:**

This article examines uptake of pictorial recall aids among caregivers of young children in Kathmandu Valley, Nepal, and Guédiawaye Department, Senegal; describes what foods/beverages are subject to recall bias in these contexts; and assesses the effect of these recall aids on estimated dietary outcomes.

**Methods:**

We used cross-sectional data from 24HR surveys that provided a recall aid to reduce caregiver recall bias. Young children’s dietary outcomes were calculated from the 24HR data. Logistic regressions were used to compare characteristics and estimated dietary outcomes of children by recall aid uptake. McNemar’s test and paired t-tests were used to compare estimated dietary outcomes in initial 24HR data with revised 24HR data that incorporated omitted items identified by the recall aid.

**Results:**

Pictorial recall aids had relatively high uptake by caregivers in both contexts. Beverages, unhealthy snacks, and fruit were most subject to recall bias. Adding omitted items identified by recall aids resulted in statistically significant changes in most dietary outcomes assessed. Children of caregivers who chose to use the recall aid had significantly different estimated dietary outcomes than children of caregivers who did not use the recall aid.

**Conclusions:**

The use of pictorial recall aids modifies 24HR results in these 2 contexts. Rigorous research is needed to fully understand the impact of recall aids on 24HR methodology and results, with an aim to improve the accuracy of dietary assessment and ultimately inform evidence-based nutrition programming, policy, and recommendations.

## Introduction

Early diets affect childhood growth and development [1] and are associated with taste preferences, dietary patterns, and health later in life [[2], [3], [4]]. Accurate measurement of young child diets is crucial to characterize dietary patterns and assess their association with nutrition- and health-related outcomes. This information is often used to inform the formulation of nutrition programs, policies, and recommendations to safeguard young child nutrition and health [[5], [6], [7], [8]]. However, accurate dietary assessment is notoriously challenging [8,9], especially among children [6,10,11].

Common dietary assessment methods for estimating food intakes at an individual level include food frequency questionnaires, quantitative 24-h recalls, estimated food records, and weighed food records [5,12,13]. Considerable trade-offs exist between methods, with the most suitable method depending on a range of factors, including the research question to be answered, whether estimates will be compared with existing data (for example, used in monitoring or to compare prevalence over time), the type of data required, the level of precision needed, time and financial constraints, and population and context characteristics [8,9,13]. The quantitative, 4-pass 24-h dietary recall (24HR) is a common method used to not only estimate the types of foods consumed but also energy and nutrient intakes [13,14]. For this method, respondents recall all foods/beverages consumed in a 24-h period and estimate portion sizes consumed for each food/beverage [15]. Although adults and adolescents can serve as respondents for their own dietary recalls, 24HR among young children typically rely on responses from the child’s primary caregiver, with other respondents solicited as necessary [6]. Strengths of the 24HR include low risk of affecting participants’ eating behavior during the period of interest, free-form response format that makes the method suitable in diverse contexts, relatively low respondent burden, and data that can be used to estimate energy and nutrient intakes [13,15,16].

The 24HR is, however, subject to limitations that can significantly affect the accuracy of its results [5,13,17]. One of these limitations is recall bias, which can manifest as forgetting consumed items, reporting items not actually consumed, or substituting consumed items with non-consumed items [18,19]. Recall bias—also referred to as “memory error”—can be a considerable source of bias in any recall-based dietary assessment method and can lead to substantial misrepresentation of diets [7,9,13,16,17,[20], [21]]. Recall bias can differ by food type, dietary pattern, and respondent characteristics, such as age, sex, or level of education. Respondents have been found to forget snack foods, drinks, and condiments (for example, sugar, salt, sauces) more often than main meals [9,22,23]. They are also more likely to forget items when a greater variety of foods/beverages were consumed and/or eating occasions were more frequent [24]. Respondent recall bias when the 24HR is used to assess the food consumption of young children may therefore be particularly problematic, given this age group’s propensity to snack, eating frequently and in small quantities. Recall bias may also be exacerbated when young children are fed by multiple people (for example, caregivers, family members, visitors, and other children).

One potential solution to mitigate recall bias in 24HR is the use of a recall aid, variously referred to as a “memory aid,” “food chart,” “pictorial chart,” “food record,” “food picture calendar,” or “food recording booklet” [[25], [26], [27], [28]]. Real-time recording of foods/beverages consumed during the 24 h of interest theoretically eliminates respondents’ reliance on memory when listing items on the day of data collection and allows multiple caregivers to record foods eaten at their time of consumption. However, to our knowledge, no prior research has measured the effect of a recall aid on 24HR results. This knowledge gap has been identified in prior research, resulting in a call for assessments of the feasibility of using recall aids in the 24HR and measurement of their effects on estimated dietary outcomes [29]. Our research, therefore, uses 24HR data from cross-sectional studies of young children in urban Nepal and peri-urban Senegal to *1*) determine the percentage of respondents who used the pictorial recall aid and the sociodemographic and contextual factors associated with its uptake; *2*) describe what foods/beverages are subject to recall bias in these contexts; and *3*) assess the effect of the recall aid on estimated indicators of infant and young child feeding (IYCF); energy, fiber, and nutrient intakes; and nutrient densities.

## Methods

This article uses data from 2 cross-sectional studies conducted by Helen Keller International during the Assessment and Research on Child Feeding project. Data were collected from February to April 2017 in Nepal and from November to December 2021 in Senegal. Both studies consisted of primary caregivers and their young children (12–23.9-mo olds in Nepal, 12–35.9-mo olds in Senegal). In Nepal, data collection took place in urban Kathmandu Valley, with a sample size of 745 caregiver-child pairs; in Senegal, data collection took place in peri-urban Guédiawaye Department, with a sample size of 724. Caregivers participated in an interviewer-administered questionnaire and a quantitative, 4-pass 24HR to identify and quantify all foods and beverages consumed by their child in the previous day. Written informed consent was collected from all caregivers. Ethical approval for both studies was granted by the London School of Hygiene and Tropical Medicine, the Nepal Health Research Council in Nepal, and the Senegalese National Ethics Committee for Health Research in Senegal.

### Development of the recall aid

Structured observations were performed in October 2016 with 10 Nepali households, to compare what trained researchers observed children were consuming with what caregivers reported children were consuming during 24HR administration. This formative research found underreporting of certain foods/beverages fed to children—namely snacks, with underreporting especially prevalent during times when the child was with someone other than the primary caregiver [30]. Pictorial recall aids were therefore integrated into each study’s protocol to reduce recall bias by asking the primary caregiver to prospectively record foods and beverages consumed by the child at the time of consumption. In Nepal, the recall aid focused on foods/beverages commonly consumed as snacks. These foods included both commercial and non-commercial foods/beverages and were identified through qualitative research conducted before the survey [31]. The recall aid was pilot tested alongside the 24HR methods to assess caregiver ease and ensure comprehension. In Nepal, the final recall aid featured pictures of 6 groups of snack foods/beverages consumed by young children in this context [32]: *1*) biscuits; *2*) candy/chocolates; *3*) fruit; *4*) milk; *5*) local porridges and cereals (jaulo or lito); and *6*) chips/salty snacks.

In Senegal, the recall aid covered all types of foods/beverages commonly consumed by young children in Dakar region, with these foods identified based on the 2018 Senegal Demographic and Health Survey [33], a 2016 dietary assessment of children aged below 24 mo in Dakar Department [34], and discussions with local informants. In Senegal, the recall aid was pilot tested among 15 caregivers of children aged 12–35.9 mo in Guédiawaye Department. Modifications based on pilot findings included increasing=image sizes for better visibility; replacing certain images with more commonly consumed and/or more recognizable items (for example, replacing images of several breastmilk substitutes with images of more recognizable brands/flavors); adding additional images to capture greater variety within food/beverage groups (for example, adding salty crackers to “salty snack foods”); moving certain images to different food/beverage groups (for example, moving peanuts from “other” to “salty snack foods”); adding new food/beverage groups (“hot beverages” and “fast food”); removing food/beverage groups (“meats, fish, seafoods, and eggs”); and modifying food/beverage groups (for example, splitting “porridges, bread, sandwiches” into 2 groups—“sandwiches” and a group listing the names of the 5 most common local porridges fed to young children—with more images added for each). In Senegal, the final recall aid featured pictures of 13 food/beverage groups: *1*) sweet snacks; *2*) salty snacks; *3*) fruit; *4*) juices/sugar-sweetened beverages; *5*) hot beverages; *6*) dairy; *7*) breastmilk substitutes; *8*) commercially produced complementary food products (instant cereals, purees); *9*) local porridges; *10*) bread/sandwiches; *11*) fast food; *12*) main meals; and *13*) other foods/beverages. The “other” category was used to note any additional items respondents wanted to include.

Both countries’ recall aids also had columns for time periods. In Nepal, this consisted of columns for each hour children were typically awake (05:00–21:00); in Senegal, this consisted of columns with text and visual representations of 3 time periods: *1*) from waking up until lunch, *2*) from lunch until dinner, and *3*) from dinner until waking up the next morning. Supplemental Figures 1 and 2 show English versions of each country’s final recall aid.

### Sampling procedure and recruitment

Probability proportional to size was used to create a representative sample of Kathmandu Valley in Nepal and Guédiawaye Department in Senegal by randomly selecting wards/neighborhoods for data collection, whereby wards/neighborhoods with a larger population had a higher probability of having one or more clusters. Detailed description of sampling procedures and recruitment is described elsewhere [26,35]. In Nepal, 78 clusters were designated within Kathmandu Valley’s 1136 municipality wards. In Senegal, 81 clusters were designated within Guédiawaye Department’s 140 neighborhoods (quartiers). A random GPS starting point within the selected ward/neighborhood was designated for each cluster. Caregiver/child pairs were recruited 2–4 d before the day of interview. Recruitment teams consisted of 2 recruiters and 1 local health agent. They conducted door-to-door recruitment using pen and paper forms, beginning at the cluster’s GPS starting point and following a systematic recruitment procedure while staying within the borders of the ward/neighborhood. Caregiver/child pairs were eligible—and caregivers were asked to provide initial consent to participate—if they were residents of the study area (that is, Kathmandu Valley or Guédiawaye Department), the child did not have a malformation that inhibited feeding, the child was within the age range of the study (aged 12 to <24 mo in Nepal, 12 to <36 mo in Senegal), and the child was not severely ill.

### Data collection procedures and tools

On the day of recruitment, caregivers who provided initial consent were given a pictorial recall aid and provided the following instructions: the date to use the recall aid (day before their day of interview); to check off the appropriate column each time the child ate or drank an item corresponding to that category, regardless of quantity; to give the recall aid to anyone looking after the child and explain how they should use it; and to give the recall aid back to the interviewer on the day of interview.

Interviews were conducted on all days of the week to remove group-level day-of-the-week effect in the 24HR data. In Nepal, interviews took place in Nepali; in Senegal, interviews took place in Wolof or French, according to respondent preference. Interviews took place in respondents’ homes to facilitate participation, as well as provide access to cooking and feeding utensils for portion size estimations. The child’s primary caregiver—defined as the person spending the most time looking after the child in a typical day—was the respondent for the questionnaire. After obtaining full informed consent, the interviewer collected the recall aid from the caregiver, setting it out of view until later use and noting if the recall aid was used in any capacity (that is, ≥1 item was checked off or encircled). The interviewer then administered the questionnaire. The questionnaire included a household roster to gather data on each household member’s age, sex, and relation to the target child, as well as questions on the demographic, health, and socioeconomic characteristics of the child, caregiver, and household. The exact number of questions depended on the activation of skip patterns but ranged from 75 to 245 questions in Nepal and 103 to 169 questions in Senegal, with the questionnaire generally taking 15–30 min to complete in Nepal and 20–40 min to complete in Senegal.

The interviewer next conducted the 24HR with the primary caregiver, soliciting information as necessary from other individuals who supervised or fed the child during the previous day or night. Respondents were first asked to list all foods/beverages—except breastmilk and water—consumed by the child, beginning when the child woke up the previous day and ending when they woke up the morning of the interview (first pass). After recording this initial list of foods/beverages, the interviewer probed extensively for any additional items to complete the first pass (for example, “Was anything added to X?;” “At the same time as the child consumed X, did they consume anything else?;” “Between the time when the child consumed X and the time when they consumed Y, did the child consume anything else, no matter how small?”). Respondents were next asked to provide food/beverage-specific details for each item consumed (second pass). They then estimated quantities consumed (third pass), using food/beverage-specific prompts, photos, or food models weighed by the interviewer on a digital scale with ±1 g precision (Nepal: Model KD-810, Tanita; Senegal: Model 1024 WHDR14, Salter). Finally, the interviewer verified all foods/beverages reported with the respondent(s) in chronological order, adding or removing foods/beverages if necessary (fourth pass).

In Nepal, the recall aid was consulted after completing the first pass of the 24HR; as the full interview (questionnaire and 24HR) typically took over 2 h and any additions from the recall aid could have been viewed as adding considerable time at the end of the interview, the recall aid was used after the first pass to reduce time burden for respondents. In Senegal, the recall aid was consulted after completing the fourth pass of the 24HR; this change from Nepal’s methodology was made to enable assessment of the recall aid’s added value to the 4-pass 24HR methodology. If the recall aid was used, the interviewer brought out the recall aid (collected before questionnaire administration), asked the respondent what each mark referred to and cross-referenced these items with the items already reported during the 24HR. If an item was not reported in the 24HR but marked on the recall aid, the interviewer confirmed with the respondent that it was indeed consumed by the child during the recall period; if consumed, it was added to the 24HR form. If an item was reported in the 24HR but not marked on the recall aid, the interviewer confirmed with the respondent that it had indeed been consumed by the child during the recall period; if not consumed, it was removed from the 24HR form. The interviewer indicated on the 24HR form if an item was added or removed due to the recall aid.

### Data quality and management

Given the complexity of 24HR administration and potential for interviewer bias, both studies had highly qualified and trained interviewers (*n =* 12 in Nepal, *n =* 10 in Senegal). All interviewers had at least high school education, significant experience collecting quantitative data (including prior experience in health or nutrition research), and native fluency in Nepali (Nepal) or Wolof and French (Senegal). Data collection training lasted 3 wk, with roughly 80% of interviewers’ time dedicated to the 24HR. The 24HR training included detailed explanation of the 4-pass method; supervised practice sessions; numerous, increasingly complex case studies debriefed by trainers; and practice 24HR with primary caregivers in non-selected wards/neighborhoods of the study area to pilot methods and select the final interviewing team.

In Nepal, questionnaires were administered on tablets using Open Data Kit and 24HR were administered on article, with 24HR data later entered into an Excel database (Microsoft Corp). In Senegal, questionnaires and 24HR were administered on tablets using CommCare, with INDDEX24 Mobile App used for the 24HR [36]. To reduce interviewer error during administration, coded constraints and skip patterns were programmed into questionnaires and comprehensive food/beverage-specific prompts were integrated into the 24HR (via a probe and portion size guide in Nepal and directly on the tablet through INDDEX24 in Senegal). Immediately after each interview, supervisors collected all documents—including recall aids—from the interviewers and checked the questionnaire and 24HR data. Interviewers returned directly to households for clarifications or corrections as necessary. Study coordinators (AMP in Nepal, AV in Senegal) were based in-country and conducted quality checks daily, including visual review of all 24HR data for completion and coherency.

### Data processing

The Household Food Insecurity Access Scale was generated from questionnaire data to determine household food security [37]. Household wealth scores were created using principal components analysis [35], and wealth quintiles generated. In Senegal, a 13-item scale (Short Form C from [38]) measured caregivers’ social desirability bias by assessing their self-reported tendency to behave in ways viewed favorably by others.

Several dietary outcomes of interest were calculated from the 24HR data, including selected WHO/UNICEF IYCF indicators; prevalence of consumption of food groups; and energy, fiber, and nutrient intakes and densities. The IYCF indicators [39] that were calculated included minimum dietary diversity, zero vegetable or fruit consumption, sweet beverage consumption, and unhealthy food consumption. Total daily energy, fiber, and nutrient intakes from non-breastmilk foods were calculated for each child using a food composition table compiled for each study, following FAO International Network of Food Data Systems guidelines [40] (details found in [26] for Nepal and [35] for Senegal). Retention factors were applied for cooked ingredients and foods when the food composition table values were for their raw equivalents, to account for nutrient loss from cooking [41]. Nutrient densities were calculated for each child by dividing total intakes for each nutrient by the child’s total energy intake of non-breastmilk foods.

### Statistical analyses

Data were cleaned and analyzed in Stata 15 (Nepal) and Stata 15.1 (Senegal). Descriptive statistics were calculated and included proportions, means ± SD for normal distributions, and medians and interquartile ranges for non-normal distributions. Bivariate cluster-controlled logistic regressions were used to compare child, caregiver, and household characteristics of caregivers who used the recall aid (“users”) with those who did not (“non-users”). Controlling for those characteristics that differed significantly (*P <* 0.05) between users and non-users, bivariate cluster-controlled logistic regressions were then used to investigate whether and to what extent dietary outcomes of users’ children differed from those of non-users’ children. Dietary outcomes investigated were *1*) prevalence of IYCF indicators and consumption of food groups, *2*) energy, fiber, and nutrient intakes, and *3*) nutrient densities. Sensitivity analyses were performed to see whether controlling for interviewer affected the statistical significance of effect sizes. Almost all results were comparable; interviewer was therefore not included as a control in reported results, but cases where significance level differed were noted in relevant tables. For caregivers who used the recall aid, McNemar’s test was used to compare the prevalence of IYCF indicators and food group consumption using *1*) the initial 24HR data before revising based on the recall aid and *2*) the revised 24HR data incorporating recall aid changes. Paired t-tests of log-transformed data were used to compare initial with revised 24HR data for energy, fiber, and nutrient intakes and nutrient densities, with Cohen’s d calculated to quantify absolute effect size of the differences between the paired groups.

## Results

In both studies, recall aid uptake was relatively high, with 639 of 745 caregivers (85.8%) using the recall aid in Nepal and 580 of 724 caregivers (80.1%) using the recall aid in Senegal. Table 1 presents key child, caregiver, and household characteristics by country, for the full sample of caregivers and by recall aid use. Nepali caregivers had higher education levels than Senegalese caregivers. Children spent time away from their caregivers at roughly twice the rate in Nepal than Senegal. Household food security was also considerably higher in Nepal than Senegal. In both countries, caregivers who used the recall aid were comparable with those who did not on most characteristics. However, in Nepal, caregivers who used the recall aid, as compared with those who did not, were significantly younger (1.6 y younger), more likely to have a formal education (88.6% compared with 79.2%), and less likely to have done paid work in the last month (28.3% compared with 38.7%). In Senegal, a significantly lower percentage of caregivers who used the recall aid, as compared with those who did not, were from food-secure households (54.8% compared with 63.9%) and spent time away from their child on the day being recalled (10.3% compared with 18.1%).

**TABLE 1:** Child, caregiver, and household characteristics, overall and by recall aid use^1^.

	Nepal				Senegal			
	Full sample (*n =* 745)	Caregiver did not use recall aid (*n =* 106)	Caregiver used recall aid (*n =* 639)	*P*	Full sample (*n =* 724)	Caregiver did not use recall aid (*n =* 144)	Caregiver used recall aid (*n =* 580)	*P*
Child								
Age (mo)	17.6 ± 3.3	17.8 ± 3.4	17.6 ± 3.3	0.656	23.2 ± 7	22.7 ± 6.8	23.4 ± 7	0.292
Caregiver								
Age (y)	29.2 ± 8.5	30.6 ± 10.2	29.0 ± 8.1	0.040	31.7 ± 9.1	31.8 ± 9.8	31.7 ± 8.9	0.848
Marital status								
Never married/lived with someone	1.1 (8)	0.9 (1)	1.1 (7)	0.893	4.1 (30)	4.9 (7)	4.0 (23)	0.619
Monogamous marriage	97.7 (728)	98.1 (104)	97.7 (624)	0.773	77.8 (563)	77.1 (111)	77.9 (452)	0.823
Polygamous marriage	0.0 (0)	0.0 (0)	0.0 (0)	1.000	14.1 (102)	13.9 (20)	14.1 (82)	0.937
Divorced/separated/widowed	1.2 (9)	0.9 (1)	1.3 (8)	0.800	4.0 (29)	4.2 (6)	4.0 (23)	0.893
Highest educational attendance								
No formal education	12.8 (95)	20.8 (22)	11.4 (73)	0.007	27.1 (196)	23.8 (34)	27.9 (162)	0.314
Primary	20.3 (151)	25.5 (27)	19.4 (124)	0.205	31.4 (227)	31.5 (45)	31.4 (182)	0.983
Middle/Secondary	52.1 (388)	44.3 (47)	53.4 (341)	0.154	32.4 (234)	32.2 (46)	32.4 (188)	0.957
Tertiary	14.9 (111)	9.4 (10)	15.8 (101)	0.120	9.1 (66)	12.6 (18)	8.3 (48)	0.054
Paid work in last 7 d	—	—	—	—	35.4 (256)	38.9 (56)	34.5 (200)	0.274
Paid work in last mo^2^	29.8 (222)	38.7 (41)	28.3 (181)	0.007	—	—	—	—
Paid work in last 12 mo	—	—	—	—	43.8 (317)	45.1 (65)	43.5 (252)	0.701
Child spent time away from them yesterday	27.0 (201)	29.3 (31)	26.6 (170)	0.566	11.9 (86)	18.1 (26)	10.3 (60)	0.008
Knows what child ate while away^3^	92.0 (185)	83.9 (26)	93.5 (159)	0.083	86.1 (74)	80.8 (21)	88.3 (53)	0.410
Social desirability score^4^	—	—	—	—	10.2 ± 1.8	10.0 ± 2.1	10.3 ± 1.8	0.107
Household								
Wealth quintile								
Poorest	20.0 (149)	21.7 (23)	19.7 (126)	0.611	20.2 (146)	18.8 (27)	20.5 (119)	0.689
Second	20.0 (149)	17.0 (18)	20.5 (131)	0.406	19.9 (144)	16.7 (24)	20.7 (120)	0.293
Middle	20.0 (149)	16.0 (17)	20.7 (132)	0.278	19.9 (144)	16.7 (24)	20.7 (120)	0.290
Fourth	20.0 (149)	18.9 (20)	20.2 (129)	0.751	20.0 (145)	25.0 (36)	18.8 (109)	0.094
Wealthiest	20.0 (149)	26.4 (28)	18.9 (121)	0.068	20.0 (145)	22.9 (33)	19.3 (112)	0.374
Food secure^5^	86.4 (644)	87.7 (93)	86.2 (551)	0.670	56.6 (410)	63.9 (92)	54.8 (318)	0.046

1 Values are % (*n*) or mean ± SD. *P* values are from bivariate, cluster-controlled logistic regressions, comparing each characteristic between caregivers who used the recall aid and those who did not use the recall aid.

2 In Nepal, caregiver was asked about paid work in the last month, whereas in Senegal caregiver was asked about paid work in the last 7 d and last 12 mo.

3 Follow-up question, only asked to caregivers if they reported that child spent time away from them yesterday.

4 Only measured in Senegal. Possible scores ranged from 0 (no social desirability bias) to 13.

5 As defined by the Household Food Insecurity Access Scale [37].

In Nepal and Senegal, respectively, there were 629 and 205 instances of a food/beverage being added due to the recall aid, with no instances of a food/beverage being removed due to the recall aid. Among caregivers who used the recall aid (Table 2), less than half had any foods/beverages added to their 24HR due to the recall aid (47.3% in Nepal, 20.7% in Senegal). In Nepal, 23.8% of caregivers who used the recall aid had 1 food/beverage added due to the recall aid, 9.6% had 2 added, 7.5% had 3 added, and 3.9% had 4 or more added. In Senegal, 11.0% had 1 food/beverage added, 6.0% had 2 added, 2.9% had 3 added, and 0.7% had 4 or more added. In both countries, unhealthy sweet snacks, fruit, unhealthy fried/salty snacks, and beverages were the types of foods/beverages most commonly added due to the recall aid. For most food/beverage categories, the Nepal study had a higher proportion of caregivers with additions due to the recall aid than the Senegal study. This difference between countries was especially pronounced for categories pictured on the Nepali recall aid (which pictured fewer categories than the Senegalese recall aid). For example, the proportion of caregivers with candy added to their 24HR data due to the recall aid was roughly 10 times higher in Nepal than Senegal.

**TABLE 2:** Percentage of caregivers using the recall aid who added a food item after reviewing the recall aid, by food group and type.

Food/beverage, by type	Nepal (*n =* 639)	Senegal (*n =* 580)
	% (*n*)^1^	
Any food/beverage type	47.3 (302)	20.7 (120)
Unhealthy sweet snack	31.3 (200)	6.9 (40)
Candy (hard candy, chocolate bar, lollipop)	22.7 (145)	2.1 (12)
Biscuit	13.2 (84)	2.1 (12)
Frozen local juice	0.0 (0)	3.3 (19)
Bakery (cake, sweet bun, donut)	0.6 (4)	0.3 (2)
Fruit	16.1 (103)	6.7 (39)
Banana	6.3 (40)	4.0 (23)
Apple	5.5 (35)	1.7 (10)
Orange/clementine	3.6 (23)	0.9 (5)
Other^2^	5.2 (33)	0.7 (4)
Unhealthy fried/salty snack	13.2 (84)	4.3 (25)
Chips/crisps	12.4 (79)	3.3 (19)
Other^3^	0.9 (6)	1.2 (7)
Beverage	9.9 (63)	5.5 (32)
Unsweetened milk (cow or buffalo)	6.9 (44)	0.0 (0)
Sweet non-fruit (tea, coffee, hot chocolate, milk with sugar)	2.0 (13)	4 (23)
Fruit drink or juice	1.1 (7)	1.7 (10)
Soda	0.0 (0)	0.2 (1)
Mixed dish	4.2 (27)	3.5 (20)
Sweet porridge or cereal^4^	1.3 (8)	2.4 (14)
Pulse or nut-based (lito, jaulo, milk and nuts)	1.3 (8)	0.0 (0)
Other^5^	1.7 (11)	1.0 (6)
Dairy (non-beverage)	0.0 (0)	2.1 (12)
Yogurt/curdled milk	0.0 (0)	1.7 (10)
Cheese	0.0 (0)	0.5 (3)
Breastmilk substitute or infant cereal	0.8 (5)	1.4 (8)
Prepared infant cereal	0.0 (0)	1.2 (7)
Breastmilk substitute	0.8 (5)	0.2 (1)
Cereal/starchy staple^6^	0.8 (5)	0.5 (3)
Pulse, nut, or seed^7^	0.0 (0)	1.0 (6)
Vegetable^7^	0.0 (0)	0.5 (3)
Egg	0.2 (1)	0.0 (0)

1 % (*n*) of caregivers who had ≥1 instance of this category being added due to the recall aid. In Nepal, the recall aid was consulted directly after the first pass of the 24-h dietary recall (24HR). In Senegal, the recall aid was consulted after all 4 passes of the 24HR.

2 In Senegal, all cases were watermelon. In Nepal, this includes pomegranate, grapes, watermelon, dates, and papaya.

3 In Senegal, all cases were fataya (deep-fried, stuffed pastry). In Nepal, all cases were instant noodles.

4 In Senegal, this includes millet porridges (thiakry, fonde) and sweet porridge-like dishes (biscuits/pasta combined with milk products and/or sugar). In Nepal, this includes cereal, biscuits, or bread with milk, as well as infant cereal with biscuits.

5 In Senegal, this includes sandwiches (baguette with filling) and pasta with sauce. In Nepal, this includes dal, kwati, and tarkari (with or without rice) and chow mein.

6 In Senegal, all cases were wheat flour baguette. In Nepal, this includes wheat bread or unsweetened bun, maize, and papad.

7 All instances of pulse, nut, or seed were peanut; all of vegetable were carrot.

Table 3 compares children’s prevalence rates of IYCF indicators and consumption of selected food groups in initial 24HR data (that is, excluding items added due to the recall aid) and revised 24HR data (that is, including items added due to the recall aid). Foods/beverages added due to the recall aid had a significant effect on prevalence rates of almost all IYCF indicators assessed. In Nepal, including items added due to the recall aid significantly changed the estimated prevalence for all indicators, except sweet beverage consumption, by 3.4–7.8 absolute percentage points. In Senegal, the effect’s magnitude was smaller, modifying the prevalence of indicators by 1.6–3.5 absolute percentage points. Including items added due to the recall aid also had a significantly positive effect on the estimated prevalence of consumption for 2 of 7 food groups in each country (dairy and other fruits/vegetables in Nepal; pulses, nuts, and seeds and other fruits/vegetables in Senegal).

**TABLE 3:** Child prevalence of WHO/UNICEF IYCF indicators and consumption of food groups, including and excluding items added due to recall aid^1^.

	Nepal (*n =* 639)			Senegal (*n =* 580)		
	Initial 24HR data	Revised 24HR data based on recall aid	Percentage point difference: B−A	Initial 24HR data	Revised 24HR data based on recall aid	Percentage point difference: B−A
	(A)	(B)	(C)	(A)	(B)	(C)
WHO/UNICEF IYCF indicators^2^						
Minimum dietary diversity^3^	70.3 (449)	73.7 (471)	3.4∗∗∗	43.6 (253)	46.4 (269)	2.8∗∗∗
Zero vegetable or fruit consumption	19.6 (125)	15.3 (98)	−4.2∗∗∗	27.8 (161)	25.2 (146)	−2.6∗∗∗
Sweet beverage consumption	43.2 (276)	43.8 (280)	0.6	68.1 (395)	69.7 (404)	1.6∗∗
Unhealthy food consumption	86.1 (550)	90.9 (581)	4.9∗∗∗	79.8 (463)	82.4 (478)	2.6∗∗∗
Unhealthy sweet food consumption	79.0 (505)	85.8 (548)	6.7∗∗∗	62.4 (362)	65.9 (382)	3.5∗∗∗
Unhealthy fried/salty food consumption	49.9 (319)	57.8 (369)	7.8∗∗∗	54.1 (314)	56.6 (328)	2.4∗∗∗
Consumption of food groups						
Cereal/starchy staples (grains, roots, tubers)^4^	99.2 (634)	99.4 (635)	0.2	99.7 (578)	99.7 (578)	0.0
Pulses (beans, peans, lentils), nuts, and seeds	89.5 (572)	89.7 (573)	0.2	30.5 (177)	31.6 (183)	1.0∗
Flesh foods (meat, fish, poultry, organ meats)	23.3 (149)	23.3 (149)	0.0	62.9 (365)	62.9 (365)	0.0
Dairy (milk, infant formula, yogurt, cheese)	78.7 (503)	80.0 (511)	1.3∗∗	89.5 (519)	89.8 (521)	0.3
Eggs	21.6 (138)	21.8 (139)	0.1	17.1 (99)	17.1 (99)	0.0
Vitamin-A-rich fruits/vegetables^5^	31.6 (202)	31.9 (204)	0.3	36.0 (209)	36.6 (212)	0.5
Other fruits/vegetables	77.5 (495)	82.6 (528)	5.2∗∗∗	59.5 (345)	62.8 (364)	3.3∗∗∗

1 In Nepal, the recall aid was consulted directly after the first pass of the 24-h dietary recall (24HR). In Senegal, the recall aid was consulted after all 4 passes of the 24HR. Values in columns A and B are % (*n*) of children. Values in Columns C are percentage point difference between proportions in Columns B and A. McNemar's test is used to compare Columns A with Columns B (paired data). Significant differences between the 2 groups concerned are shown in Columns C when ∗∗∗*P <* 0.001,∗∗*P <* 0.01, ∗*P <* 0.05.

2 As defined in the WHO/UNICEF 2021 guidelines [39].

3 % of children who consumed foods and beverages from ≥5 out of 8 defined food groups during the previous day. The 8 food groups include the 7 listed in the table, plus breastmilk.

4 Includes bread products and cereal-based commercially produced complementary foods. Excludes sweet and salty confectionary (e.g., cakes, biscuits).

5 Provide ≥120 retinol equivalents (RE) or ≥60 retinol activity equivalents (RAE), per 100 g edible portion (e.g., papaya, carrot).

Children’s median intakes of energy, fiber, and all 16 nutrients assessed were significantly higher when 24HR data included items added due to the recall aid (Table 4). In Nepal, median intakes were 3.7%–13.2% higher in revised 24HR data as compared with initial 24HR data, whereas in Senegal they were 0.8%–5.5% higher. Nutrient density differences were generally of smaller magnitude than intakes and were both higher and lower in revised 24HR data compared with initial 24HR data (as opposed to intakes, which were all higher in revised 24HR data). In Nepal, the significant differences for nutrient densities ranged from −1.0% to 6.2%: 8 of 17 densities assessed were significantly higher when the 24HR data included foods/beverages added due to the recall aid (fiber, total fat, saturated fat, total sugar, sodium, vitamins B12 and C, and folate), whereas 2 were significantly lower (protein and zinc). In Senegal, significant differences ranged from −0.9% to 3.9%: 4 of 17 densities assessed were significantly higher when the 24HR data included foods/beverages added due to the recall aid (total sugar, vitamin C, niacin, folate), whereas 4 were significantly lower (protein, total fat, sodium, and vitamin A). Despite widespread statistical significance, Cohen’s d values indicate relatively small effect sizes, ranging from 0.16 to 0.53 SDs for intakes and 0.01 to 0.37 SDs for densities.

**TABLE 4:** Child total energy, fiber, and nutrient intakes and densities of non-breastmilk foods, including and excluding items added due to recall aid^1^.

	Nepal (n = 639)			Senegal (*n =* 580)		
	Initial 24HR data	Revised 24HR data based on recall aid	Percent difference in medians (effect size)	Initial 24HR data	Revised 24HR data based on recall aid	Percent difference in medians (effect size)
	(A)	(B)	(C)	(A)	(B)	(C)
Intakes (per d)						
Total energy intake (kcal)	588 (426–816)	623 (454–860)	6.0∗∗∗ (0.53)	1072 (752–1392)	1093 (778–1422)	2.0∗∗∗ (0.34)
Fiber (g)	4.7 (2.6–7.2)	5.0 (3.0–7.5)	6.5∗∗∗ (0.41)	7.5 (4.9–10.7)	7.8 (5.1–11.2)	3.1∗∗∗ (0.32)
Protein (g)	17.1 (11.8–25.7)	18.2 (12.3–26.7)	6.7∗∗∗ (0.44)	24.6 (17.4–33.4)	25.4 (17.6–34.2)	3.0∗∗∗ (0.28)
Total fat (g)	18.2 (11.9–27.6)	19.2 (12.5–29.8)	5.5∗∗∗ (0.47)	32.6 (19.6–44.1)	33.3 (19.9–45.2)	1.9∗∗∗ (0.23)
Saturated fat (g)	7.9 (4.6–12.2)	8.5 (4.9–13.2)	6.8∗∗∗ (0.45)	10.1 (6.5–14.6)	10.3 (6.5–14.9)	1.9∗∗∗ (0.24)
Total sugar (g)	24.2 (15.0–38.1)	27.4 (16.9–41.0)	13.2∗∗∗ (0.52)	62.8 (43.7–89.2)	66.2 (44.7–92.2)	5.5∗∗∗ (0.32)
Sodium (mg)	215 (131–341)	238 (149–379)	10.5∗∗∗ (0.39)	1102 (718–1603)	1134 (730–1651)	3.0∗∗∗ (0.27)
Calcium (mg)	230 (97–412)	243 (113–435)	5.7∗∗∗ (0.33)	361 (218–552)	364 (218–563)	0.8∗∗∗ (0.21)
Iron (mg)	3.1 (1.9–4.8)	3.3 (2.1–5.0)	6.9∗∗∗ (0.37)	7.5 (4.9–10.3)	7.6 (5.0–10.7)	1.7∗∗∗ (0.25)
Zinc (mg)	2.35 (1.52–3.57)	2.47 (1.60–3.75)	5.1∗∗∗ (0.43)	3.83 (2.72–5.32)	3.88 (2.77–5.40)	1.3∗∗∗ (0.21)
Vitamin C (mg)	11.8 (4.1–27.5)	13.1 (4.8–30.2)	11.7∗∗∗ (0.24)	34.2 (18.4–57.7)	35.2 (19.6–62.3)	3.2∗∗∗ (0.23)
Thiamin (mg)	0.31 (0.20–0.51)	0.33 (0.20–0.53)	5.3∗∗∗ (0.35)	0.41 (0.28–0.62)	0.43 (0.28–0.63)	4.8∗∗∗ (0.26)
Riboflavin (mg)	0.56 (0.28–0.10)	0.60 (0.31–1.01)	7.6∗∗∗ (0.35)	0.65 (0.44–0.98)	0.66 (0.45–1.00)	1.3∗∗∗ (0.24)
Niacin (mg)	3.59 (2.14–6.04)	3.88 (2.27–6.28)	8.2∗∗∗ (0.33)	4.66 (2.82–6.62)	4.79 (2.94–6.73)	3.0∗∗∗ (0.23)
Vitamin B-6 (mg)	0.35 (0.21–0.55)	0.37 (0.22–0.56)	4.8∗∗∗ (0.30)	0.63 (0.40–0.92)	0.66 (0.42–0.95)	5.1∗∗∗ (0.27)
Vitamin B-12 (μg)	0.65 (0.26–1.15)	0.71 (0.31–1.24)	9.2∗∗∗ (0.35)	1.34 (0.74–2.14)	1.38 (0.75–2.21)	2.9∗∗∗ (0.16)
Folate (μg)	51.6 (28.4–81.6)	55.4 (32.7–87.5)	7.4∗∗∗ (0.41)	126.2 (75.6–177.0)	129.7 (78.4–181.0)	2.7∗∗∗ (0.30)
Vitamin A (RAE) (μg)	115.0 (51.9–210.4)	119.3 (53–214.4)	3.7∗∗∗ (0.27)	484.3 (287.0–731.2)	490.3 (288.6–732.6)	1.2∗∗∗ (0.19)
Densities (per 100 kcal)						
Fiber (g)	0.8 (0.5–1.1)	0.8 (0.6–1.1)	0.7∗∗ (0.05)	0.7 (0.6–0.9)	0.7 (0.6–0.9)	1.0 (0.03)
Protein (g)	2.9 (2.5–3.5)	2.9 (2.4–3.4)	−2.0∗∗∗ (0.34)	2.3 (2.0–2.7)	2.3 (2.0–2.7)	−0.4∗ (0.12)
Total fat (g)	3.2 (2.6–3.8)	3.2 (2.6–3.8)	0.0∗ (0.01)	3.0 (2.5–3.6)	3.0 (2.5–3.5)	−0.2∗ (0.11)
Saturated fat (g)	1.3 (1.0–1.8)	1.4 (1.0–1.7)	1.5∗∗∗ (0.12)	1.0 (0.8–1.2)	1.0 (0.8–1.2)	−0.5 (0.07)
Total sugar (g)	4.1 (3.1–5.4)	4.4 (3.3–5.6)	6.2∗∗∗ (0.37)	6.2 (4.9–7.6)	6.3 (5.0–7.7)	1.5∗∗∗ (0.21)
Sodium (mg)	36 (26–51)	38 (27–53)	3.8∗∗∗ (0.22)	109 (83–133)	108 (84–131)	−0.9∗∗ (0.15)
Calcium (mg)	39 (21–59)	40 (21–59)	0.8 (0.02)	35 (21–53)	35 (21–53)	0.4 (0.02)
Iron (mg)	0.5 (0.4–0.7)	0.5 (0.4–0.7)	0.3 (0.12)	0.7 (0.6–0.8)	0.7 (0.6–0.8)	−0.4 (0.02)
Zinc (mg)	0.40 (0.31–0.49)	0.39 (0.31–0.48)	−1.0∗∗∗ (0.33)	0.36 (0.30–0.44)	0.36 (0.3–0.43)	−0.1 (0.02)
Vitamin C (mg)	2 (0.7–4.3)	2.1 (0.8–4.4)	5.5∗∗∗ (0.10)	3.2 (2.0–5.5)	3.3 (2.0–5.5)	3.9∗∗ (0.07)
Thiamin (mg)	0.05 (0.04–0.07)	0.05 (0.04–0.07)	−0.6 (0.09)	0.04 (0.03–0.05)	0.04 (0.03–0.05)	0.3 (0.04)
Riboflavin (mg)	0.10 (0.06–0.14)	0.10 (0.06–0.14)	0.5 (0.08)	0.06 (0.05–0.09)	0.06 (0.05–0.09)	0.5 (0.04)
Niacin (mg)	0.61 (0.45–0.81)	0.61 (0.46–0.80)	−0.8 (0.07)	0.41 (0.31–0.54)	0.41 (0.32–0.54)	0.6∗ (0.05)
Vitamin B-6 (mg)	0.06 (0.05–0.07)	0.06 (0.05–0.07)	0.3 (0.09)	0.06 (0.05–0.08)	0.06 (0.05–0.08)	0.9 (0.02)
Vitamin B-12 (μg)	0.11 (0.06–0.17)	0.11 (0.06–0.16)	1.6∗ (0.02)	0.13 (0.08–0.20)	0.13 (0.08–0.21)	1.2 (0.04)
Folate (μg)	8.5 (5.9–11.3)	8.6 (6.1–11.3)	1.2∗∗ (0.04)	11.6 (8.8–14.7)	11.7 (8.9–14.7)	1.0∗ (0.06)
Vitamin A (RAE) (μg)	18.7 (10.3–28.7)	18.1 (10.4–28)	−2.7 (0.16)	46.6 (33.1–60.2)	46.3 (32.9–59.7)	−0.5∗∗∗ (0.21)

Abbreviations: 24HR, 24-h dietary recall; RAE, retinol activity equivalent.

1 In Nepal, the recall aid was consulted directly after the first pass of the 24HR. In Senegal, the recall aid was consulted after all 4 passes of the 24HR. Values in Columns A and B are median (interquartile range). The first values in Columns C are percent difference of medians, calculated as: B − A/A × 100. Log-transformed data with paired t-tests is used to compare Column A with Column B (paired data); significant differences between the 2 groups concerned are shown in Columns C when ∗∗∗*P <* 0.001, ∗∗*P <* 0.01, ∗*P <* 0.05. Absolute Cohen’s d values (effect size of the differences between the paired groups, in SD units) are displayed in brackets in Column C.

Supplemental Tables 1 and 2 compare the above dietary outcomes using 24HR data for children whose caregiver did not use the recall aid (Columns A) with initial 24HR data (that is, before adjusting the results based on the recall aid) for children whose caregiver did (Columns B). In both countries, prevalence of unhealthy sweet food consumption was significantly higher for children whose caregiver chose to use the recall aid as compared with children whose caregiver did not. In each country, median energy, fiber, and nutrient intakes were significantly higher for children whose caregiver chose to use the recall aid for 13 of 18 intakes assessed, although in Nepal they were also lower for 2 intakes assessed.

## Discussion

Recall bias can affect the accuracy of 24HR data and lead to diet misrepresentation [7,9,13,16,17,[20], [21]]. Recall bias may be particularly problematic when using the 24HR to estimate the dietary intakes of young children, because their recalls cannot be done through self-reporting. Young children may be fed by multiple individuals and eat frequently and in small quantities, making remembering all items they consumed more challenging [9,[22], [23], [24]]. To our knowledge, the effect of a recall aid on 24HR results has not been previously assessed, although there is broader literature on other approaches, including use of digital tools, to improve the accuracy of dietary assessments [[42], [43], [44], [45]]. In Kathmandu Valley, Nepal, and Guédiawaye Department, Senegal, pictorial recall aids had relatively high uptake by the caregivers, indicating they are feasible to use in these urban and semi-urban contexts. Beverages, unhealthy snacks, and fruit were most commonly added to 24HR data after consulting the recall aid. Adding these omitted items resulted in statistically significant changes in most dietary outcomes assessed.

In both studies, over 80% of caregivers used the recall aid in some capacity. A study in rural Tanzania found even higher uptake, ranging from 89.3% to 96.4% over 8 monthly household 24-h food recalls [29]. This high uptake in 3 different contexts indicates that pictorial recall aid use is feasible, including in settings where user education is low: over a quarter of caregivers using the recall aid in Senegal had no formal education and 21.9% of respondents in Tanzania had no formal education [29]. Multiple factors likely influence within-population and between-population uptake of a pictorial dietary recall aid, including tool design, clarity of explanation by the research team, and user characteristics (for example, age, literacy, motivation, time, proximity to child during the day).

Although recall aid uptake was measured binarily in Nepal and Senegal, research staff in both countries reported anecdotally that caregivers who used the recall aid had diverse comprehension and use of the tool. Some caregivers fully completed their recall aid and shared with interviewers that they carefully explained its use to others responsible for the child. Other caregivers marked one or very few items compared with the number reported during their 24HR or used the tool incorrectly by marking items consumed outside of the recall period. Caregivers shared a number of challenges with interviewers, including trouble deciding where to classify a given food/beverage, needing assistance filling out the recall aid due to illiteracy or unfamiliarity holding a writing implement, difficulties keeping the recall aid clean and intact while still close at hand (in several cases, their young child ripped or spilled on the recall aid), and/or difficulties explaining the recall aid to others looking after the child and encouraging them to use it. Future research investigating how recall aids are understood and used by respondents in different contexts is needed to generate recommendations for tool design and explanation.

In this research, fruit, unhealthy sweet or fried/salty snacks, and beverages were most commonly added due to the recall aid in both countries. This is unsurprising in Nepal because the recall aid focused on foods/beverages commonly consumed as snacks. The Senegal recall aid covered all types of foods/beverages commonly consumed by young children in Dakar region, and the types of foods/beverages added due to the recall aid are largely consistent with prior literature from other contexts. The USDA identified beverages, sweets, savory snacks, fruits, vegetables, cheese, and breads as common “forgotten foods” in multiple pass 24HR [22,46]. A comparison of camera-assisted 24HR to doubly labeled water among New Zealand adults found that snacks (healthy and unhealthy), condiments, and beverages were the most commonly forgotten items in 24HR [47]. This evidence underscores the importance of ensuring that recall aids include categories of foods/beverages eaten irregularly and/or in small quantities. This consideration is particularly salient for the age group considered in this study, children aged 12–35.9 mo, because most foods/beverages are consumed in small quantities due to their limited gastric capacity. Regardless of age group, recall aid development must be context specific. Country-adapted food/beverage lists, such as the general population and IYCF questionnaires prepared by the Global Diet Quality Project [48], could be used to inform image selection for studies developing recall aids, with due consideration of regional dietary variation within countries and populations.

In this research, the omitted foods/beverages added due to recall aids had implications for most dietary outcomes assessed, including significantly higher prevalence for most IYCF indicators, significantly lower zero vegetable or fruit consumption, and significantly higher median energy, fiber, and nutrient intakes. These findings complement the Tanzanian study, which found that incorporating omitted items from recall aids resulted in higher reported household dietary diversity and consumption prevalence of foods/beverages such as fruit, soft drinks, eggs, fish, beans, Bambara nuts, and ginger [29]. Logically, densities in our research changed less than intakes when omitted foods/beverages were added, because densities were scaled to total energy intakes (which also changed when adding omitted items). However, densities were still significantly different for roughly half of nutrients assessed, due to commonly omitted items having different nutrient profiles than included ones. For example, in both countries, total sugar was significantly denser in revised 24HR data than initial 24HR data, due to high sugar density in commonly omitted items (for example, unhealthy sweet snacks, fruit, sweet beverages). However, differences in intakes and densities were generally small in magnitude, so practical significance may be limited, depending on the context. In 2021, the WHO [39] released a suite of updated indicators for assessing IYCF practices, including new indicators of unhealthy food and beverage consumption and an indicator of zero vegetable or fruit consumption. Given the global focus on both the healthy and unhealthy aspects of young children’s diets, the findings from this study indicate that the integration of a recall aid in 24HR can result in varying prevalence estimates, potentially having implications for comparability of results across studies (if some studies use recall aids and some do not) as well as for program design and policy, depending on the level of precision needed in dietary estimates.

Because the recall aid was consulted after the first pass of the 24HR in Nepal and after the final (fourth) pass in Senegal, it is unsurprising that the Nepal study had a higher proportion of caregivers with additions due to the recall aid (both overall and for many food/beverage types), as well as generally larger magnitude differences than Senegal when comparing dietary outcomes in initial 24HR data with those in revised 24HR data. In 24HR administration, foods/beverages may be added or removed in any pass and the fourth pass specifically acts as a final review with the respondent, to determine whether any foods/beverages need to be added or removed. Interviewers in Senegal reported that it was relatively common for foods/beverages to be added during the second, third, and/or fourth passes, generally due to caregivers remembering forgotten items when discussing the child’s consumption in detail. If the recall aid had been consulted after the first pass in Senegal, as it was in Nepal, items added due to this spontaneous caregiver recall in the second, third, or fourth passes may rather have been added due to the recall aid. The Nepal study therefore may overstate the recall aid’s effect on omissions and dietary outcomes. Consulting the recall aid after the first pass could theoretically reduce time burden on participants and interviewers, because adding new foods/beverages in later passes of the 24HR tends to take more time than adding them during the first pass. Other studies may consider consulting the recall aid before the fourth pass, so that the fourth pass still acts as the final review of consumption over the 24-h period. Future research could study optimal timing of recall aid consultation within 24HR administration, considering factors such as interview flow and time burden. Although the Nepal study may overstate the recall aid’s effect in 24HR, it provides an indication of the effect magnitude that might be seen if a recall aid were added to single-pass dietary assessment methods, such as open dietary recalls used in many large-scale surveys.

For prevalence of IYCF indicators and food group consumption, as well as energy, fiber, and nutrient intakes, there were many significant differences between children whose caregiver chose to use the recall aid and children whose caregiver did not. These differences were generally in the direction of higher reported consumption for children whose caregiver used the recall aid, even in the initial 24HR data (that is, before foods/beverages were added due to the recall aid). The reasons for differential outcomes between users and non-users are unclear but could be due to several factors, which are not mutually exclusive. First, differences in characteristics between caregivers may have affected recall aid uptake as well as reported or actual consumption. For example, in Nepal, recall aid uptake was associated with level of education, which can influence diet quality [34,49]; in Senegal, it was associated with whether a caregiver was away from their child, which could cause underreporting. Characteristics not measured in the studies—such as caregiver motivation, time, and/or comprehension of instructions—could also explain differences in both recall aid uptake and reported consumption. Second, filling out the recall aid may have increased caregivers’ awareness or ability to remember their child’s consumption during the recall period, as has been reported previously in rural Tanzania [29]. Finally, images on the recall aid may have prompted caregivers using the recall aid to feed (or report feeding) their child differently than usual, although social desirability scores in Senegal indicate no difference in caregivers’ tendency to perform “as desired” according to recall aid use. Social desirability was not measured in Nepal. Respondent bias is well documented for food record dietary assessment, which involves respondents’ real-time recording of foods/beverages consumed and quantities [16,50,51]. Although our research cannot identify which, if any, of these 3 factors caused differential dietary outcomes by recall aid use, it does indicate that including a recall aid in 24HR methodology could introduce a differential bias in outcomes if some participants do not use it.

This research has 3 key limitations. The most important limitation is that, due to budgetary and time constraints associated with our relatively large sample sizes, we did not employ multiple dietary assessment methods in either country. We are thus unable to compare dietary outcomes from recall aid-assisted 24HR to non-recall-aid-assisted 24HR for the same child or to compare recall-aid-assisted 24HR to non-recall reference method(s), such as doubly labeled water, biomarkers, or weighed food records. As such, although our research indicates that recall aids affect 24HR results, it cannot determine whether they improved accuracy (by improving caregiver recall and capturing foods/beverages consumed that would have been forgotten) or compromised it (by biasing behavior during the recall period and/or leading caregivers to over-report consumption). Given that recall aids are commonly used in 24HR methodology [15,[25], [26], [27], [28]], future research—including validation studies in different contexts—is critical to determine whether recall aids ultimately improve the accuracy of 24HR results and should be recommended for use in 24HR. Second, although uptake of recall aids is assessed in this paper, respondents’ comprehension and adherence to instructions are not assessed, as noted above. If recall aids are found to strengthen accuracy of dietary assessment, qualitative research could provide rich insights on ways of improving recall aid uptake, use, and comprehension in different contexts. Lastly, direct comparison of Nepal and Senegal results is limited by key differences in the 2 studies, namely different child age ranges and diets, different recall aid design, and—most importantly—different timing of recall aid consultation during 24HR administration, as noted above.

There is considerable potential value in recall aids to reduce recall bias, a known limitation of 24HR. Recall aids must, however, be thoughtfully developed, tested, and validated to determine whether they ultimately improve the accuracy of dietary assessments. We call for rigorous quantitative and qualitative research in various contexts to provide necessary data on the impact of recall aids on 24HR methodology and results, with an aim to improve the accuracy of 24HR dietary assessment and ultimately inform evidence-based nutrition programming, policy, and recommendations.

Recall bias is also a concern in other recall-based dietary assessment methods. If recall aids are found to greatly improve the accuracy of 24HR dietary data, future research may wish to examine their impact on other recall-based methods. However, given their inclusion necessitates a household visit before data collection, recall aids may not be feasible for large-scale, multi-topic surveys that use list-based or open recalls to inform global nutrition knowledge (for example, Demographic and Health Survey, Multiple Indicator Cluster Survey).

## Author contributions

The authors’ responsibilities were as follows – AMP, ELF: designed research in Nepal and Senegal, with input from NS (Nepal) and AV, NYS, and RK (Senegal); AMP: conducted research in Nepal and AV: conducted research in Senegal; AV: analyzed data and wrote the article, with input from AMP and ELF; AV: had primary responsibility for final content; and all authors: read and approved the final version.

## Conflict of interest

The authors report no conflicts of interest.

## Funding

This work was supported, in whole or in part, by the Bill & Melinda Gates Foundation [OPP1190179]. Under the grant conditions of the Foundation, a Creative Commons Attribution 4.0 Generic License has already been assigned to the Author Accepted Manuscript version that might arise from this submission. The supporting source had no involvement or restrictions regarding submission for publication.

## Data availability

Data described in the manuscript, code book, and analytic code will be made available upon request.
