# Supplementary material for: The Use of Pictorial Recall Aids Modifies Dietary Assessment Results: Experiences from Quantitative 24-hour Dietary Recalls of Young Children in Nepal and Senegal
Source: Curr Dev Nutr. 2024 Aug 30;9(Suppl 1):104452. doi: 10.1016/j.cdnut.2024.104452 (PMC12125692; doi:10.1016/j.cdnut.2024.104452)
Supplement: multimedia component 1 [file mmc1.docx]

| **Supplementary Table 1: Child prevalence of WHO/UNICEF IYCF indicators and consumption of food groups,**  **by recall aid use ^1^** | | | | | | | | | | | | |
| --- | --- | --- | --- | --- | --- | --- | --- | --- | --- | --- | --- | --- |
|  | | **Nepal** | | | | | **Senegal** | | | | |  |
|  | | **Caregiver did not use recall aid (n=106)** | **Initial 24HR data (n=639)** | | **Percentage point difference: B-A** | | **Caregiver did not use recall aid (n=144)** | **Initial 24HR data (n=580)** | | **Percentage point difference: B-A** | |  |
|  | | (A) | (B) | | (C) | | (A) | (B) | | (C) | |  |
| *WHO/UNICEF IYCF indicators*^2^ | |  |  | |  | |  |  | |  | |  |
| Minimum dietary diversity^3^ | | 66.0 (70) | 70.3 (449) | | 4.2^6^ | | 31.9 (46) | 43.6 (253) | | 11.7** | |  |
| Zero vegetable or fruit consumption | | 22.6 (24) | 19.6 (125) | | -3.1 | | 40.3 (58) | 27.8 (161) | | -12.5*** | |  |
| Sweet beverage consumption | | 56.6 (60) | 43.2 (276) | | -13.4**^7^ | | 63.2 (91) | 68.1 (395) | | 4.9 | |  |
| Unhealthy food consumption | | 87.7 (93) | 86.1 (550) | | -1.7 | | 75.7 (109) | 79.8 (463) | | 4.1* | |  |
| Unhealthy sweet food consumption | | 78.3 (83) | 79.0 (505) | | 0.7* | | 54.2 (78) | 62.4 (362) | | 8.2** | |  |
| Unhealthy fried/salty food consumption | | 47.2 (50) | 49.9 (319) | | 2.8 | | 45.8 (66) | 54.1 (314) | | 8.3* | |  |
| *Consumption of food groups* | |  |  | |  | |  |  | |  | |  |
| Cereal/starchy staples (grains, roots, tubers)^4^ | | 95.3 (101) | 99.2 (634) | | 3.9** | | 100.0 (144) | 99.7 (578) | | -0.3 | |  |
| Pulses (beans, peans, lentils), nuts, and seeds | | 83.0 (88) | 89.5 (572) | | 6.5* | | 21.5 (31) | 30.5 (177) | | 9.0** | |  |
| Flesh foods (meat, fish, poultry, organ meats) | | 26.4 (28) | 23.3 (149) | | -3.1 | | 56.9 (82) | 62.9 (365) | | 6.0 | |  |
| Dairy (milk, infant formula, yogurt, cheese) | | 69.8 (74) | 78.7 (503) | | 8.9** | | 89.6 (129) | 89.5 (519) | | -0.1 | |  |
| Eggs | | 24.5 (26) | 21.6 (138) | | -2.9 | | 16.0 (23) | 17.1 (99) | | 1.1 | |  |
| Vitamin-A-rich fruits/vegetables^5^ | | 31.1 (33) | 31.6 (202) | | 0.5 | | 37.5 (54) | 36.0 (209) | | -1.5 | |  |
| Other fruits/vegetables | | 73.6 (78) | 77.5 (495) | | 3.9 | | 42.4 (61) | 59.5 (345) | | 17.1*** | |  |
| ^1^ In Nepal, the recall aid was consulted directly after the first pass of the 24HR. In Senegal, the recall aid was consulted after all four passes of the 24HR. Values in Columns A and B are % (n) of children. Values in Columns C are percentage point difference between proportions in Columns B and A. Bivariate, cluster-controlled logistic regressions are used to compare caregivers in Column A with those Column B (unpaired data), controlling for variables with p<0.05 in Table 1 (Nepal: caregiver age, no formal education, paid work in the last month; Senegal: child spent time away from caregiver yesterday; household is food secure). Significant differences between the two groups concerned are shown in Columns C when *** p<0.001,** p<0.01, * p<0.05 for the recall aid use coefficient.  ^2^ As defined in the WHO/UNICEF 2021 guidelines (40). | | | | | | | | | | | |  |
| ^3^ % of children who consumed foods and beverages from at least five out of eight defined food groups during the previous day. The eight food groups include the seven listed in the table, plus breastmilk. | | | | | | | | | | | |  |
| ^4^ Includes bread products and cereal-based commercially produced complementary foods. Excludes sweet and salty confectionary (e.g., cakes, biscuits). | | | | | | | | | | | |  |
| ^5^ Provide at least 120 retinol equivalents (RE) or at least 60 retinol activity equivalents (RAE), per 100 g edible portion (e.g., papaya, carrot).  ^6^ When adding in controls for interviewer during sensitivity analyses, effect size is no longer statistically significant at p<0.05.  ^7^ When adding in controls for interviewer during sensitivity analyses, effect size becomes statistically significant at p<0.05 rather than p<0.01. | | | | | | | | | | | |  |
| **Supplementary Table 2: Child total energy, fiber, and nutrient intakes and densities of non-breastmilk foods, by recall aid use ^1^** | | | | | | | | | | | | |
|  | **Nepal** | | | | | **Senegal** | | | | | | |
|  | **Caregiver did not use recall aid (n=106)** | **Initial 24HR data (n=639)** | | **Percent difference in medians** | | **Caregiver did not use recall aid (n=144)** | | | **Initial 24HR data (n=580)** | | **Percent difference in medians** | |
|  | (A) | (B) | | (C) | | (A) | | | (B) | | (C) | |
| **Intakes (per day)** |  |  | |  | |  | | |  | |  | |
| Total energy intake, kcal | 554 (333-834) | 588 (426-816) | | 6.2** | | 1011 (605-1295) | | | 1072 (752-1392) | | 6.0** | |
| Fiber, g | 4.0 (2.2-6.5) | 4.7 (2.6-7.2) | | 16.1*^3^ | | 6.7 (3.8-9.5) | | | 7.5 (4.9-10.7) | | 12.0** | |
| Protein, g | 17.0 (8.3-27.2) | 17.1 (11.8-25.7) | | 0.4* | | 22.5 (15.5-31.9) | | | 24.6 (17.4-33.4) | | 9.5* | |
| Total fat, g | 16.3 (7.4-29.6) | 18.2 (11.9-27.6) | | 11.7** | | 27.1 (17.0-41.3) | | | 32.6 (19.6-44.1) | | 20.2* | |
| Saturated fat, g | 6.2 (3.2-12.3) | 7.9 (4.6-12.2) | | 27.9** | | 9.3 (6.2-13.9) | | | 10.1 (6.5-14.6) | | 8.1 | |
| Total sugar, g | 22.7 (10.8-39.8) | 24.2 (15.0-38.1) | | 6.7*** | | 61.4 (34.4-84.6) | | | 62.8 (43.7-89.2) | | 2.2** | |
| Sodium, mg | 205 (122-383) | 215 (131-341) | | 4.9** | | 976 (571-1494) | | | 1102 (718-1603) | | 12.9** | |
| Calcium, mg | 179 (49-373) | 230 (97-412) | | 28.6*** | | 371 (215-540) | | | 361 (218-552) | | -2.6 | |
| Iron, mg | 3.3 (1.8-5.4) | 3.1 (1.9-4.8) | | -6.0 | | 7.5 (4.2-9.6) | | | 7.5 (4.9-10.3) | | 0.1* | |
| Zinc, mg | 2.16 (1.05-4.01) | 2.35 (1.52-3.57) | | 8.9* | | 3.74 (2.68-5.19) | | | 3.83 (2.72-5.32) | | 2.4 | |
| Vitamin C, mg | 8.9 (2.8-21.5) | 11.8 (4.1-27.5) | | 32.1 | | 26.5 (14.8-55.8) | | | 34.2 (18.4-57.7) | | 28.7** | |
| Thiamin, mg | 0.31 (0.14-0.46) | 0.31 (0.20-0.51) | | -0.2** | | 0.37 (0.26-0.54) | | | 0.41 (0.28-0.62) | | 9.5* | |
| Riboflavin, mg | 0.45 (0.17-0.89) | 0.56 (0.28-0.10) | | 23.2*** | | 0.65 (0.39-0.95) | | | 0.65 (0.44-0.98) | | 0.8 | |
| Niacin, mg | 3.75 (1.56-6.01) | 3.59 (2.14-6.04) | | -4.2* | | 4.06 (2.15-5.67) | | | 4.66 (2.82-6.62) | | 14.8** | |
| Vitamin B-6, mg | 0.33 (0.17-0.50) | 0.35 (0.21-0.55) | | 9.0** | | 0.52 (0.32-0.74) | | | 0.63 (0.40-0.92) | | 20.5*** | |
| Vitamin B-12, µg | 0.58 (0.15-1.22) | 0.65 (0.26-1.15) | | 11.7 | | 1.36 (0.65-2.08) | | | 1.34 (0.74-2.14) | | -1.3 | |
| Folate, µg | 49.2 (20.7-89.9) | 51.6 (28.4-81.6) | | 4.9* | | 108.6 (60.2-165.2) | | | 126.2 (75.6-177.0) | | 16.3** | |
| Vitamin A (RAE)^2^, µg | 90.7 (23.7-219.5) | 115.0 (51.9-210.4) | | 26.9* | | 389.2 (217.4-642.6) | | | 484.3 (287.0-731.2) | | 24.4* | |
| **Densities (per 100 kcal)** |  |  | |  | |  | | |  | |  | |
| Fiber, g | 0.7 (0.5-1.1) | 0.8 (0.5-1.1) | | 12.4 | | 0.7 (0.5-0.8) | | | 0.7 (0.6-0.9) | | 5.1 | |
| Protein, g | 3.1 (2.4-3.6) | 2.9 (2.5-3.5) | | -4.4 | | 2.4 (2.1-2.7) | | | 2.3 (2.0-2.7) | | -2.9 | |
| Total fat, g | 3.1 (2.4-3.8) | 3.2 (2.6-3.8) | | 1.9 | | 3.0 (2.5-3.5) | | | 3.0 (2.5-3.6) | | 0.1 | |
| Saturated fat, g | 1.2 (0.8-1.7) | 1.3 (1.0-1.8) | | 12.4* | | 1.0 (0.8-1.3) | | | 1.0 (0.8-1.2) | | -5.6 | |
| Total sugar, g | 4.2 (3.1-5.9) | 4.1 (3.1-5.4) | | -0.7 | | 6.3 (5.0-7.6) | | | 6.2 (4.9-7.6) | | -0.5 | |
| Sodium, mg | 41 (29-57) | 36 (26-51) | | -12.3 | | 112 (80-135) | | | 109 (83-133) | | -2.8 | |
| Calcium, mg | 35 (14-57) | 39 (21-59) | | 12.7** | | 41 (26-57) | | | 35 (21-53) | | -15.5 | |
| Iron, mg | 0.6 (0.4-0.8) | 0.5 (0.4-0.7) | | -12.7 | | 0.7 (0.6-0.9) | | | 0.7 (0.6-0.8) | | -0.4 | |
| Zinc, mg | 0.40 (0.29-0.50) | 0.40 (0.31-0.49) | | -1.1 | | 0.40 (0.35-0.46) | | | 0.36 (0.30-0.44) | | -8.9** | |
| Vitamin C, mg | 1.8 (0.7-3.8) | 2 (0.7-4.3) | | 7.2 | | 2.9 (1.8-5.2) | | | 3.2 (2.0-5.5) | | 11.2 | |
| Thiamin, mg | 0.05 (0.04-0.07) | 0.05 (0.04-0.07) | | -4.1* | | 0.04 (0.03-0.05) | | | 0.04 (0.03-0.05) | | -1.6 | |
| Riboflavin, mg | 0.08 (0.05-0.12) | 0.10 (0.06-0.14) | | 13.0** | | 0.07 (0.05-0.09) | | | 0.06 (0.05-0.09) | | -5.9 | |
| Niacin, mg | 0.66 (0.45-0.85) | 0.61 (0.45-0.81) | | -6.9 | | 0.36 (0.28-0.53) | | | 0.41 (0.31-0.54) | | 13.7 | |
| Vitamin B-6, mg | 0.06 (0.04-0.07) | 0.06 (0.05-0.07) | | 4.2 | | 0.05 (0.04-0.07) | | | 0.06 (0.05-0.08) | | 10.2** | |
| Vitamin B-12, µg | 0.10 (0.04-0.18) | 0.11 (0.06-0.17) | | 7.6 | | 0.13 (0.09-0.21) | | | 0.13 (0.08-0.20) | | -3.4 | |
| Folate, µg | 8.4 (5.9-11.6) | 8.5 (5.9-11.3) | | 1.7 | | 11.2 (7.8-14.6) | | | 11.6 (8.8-14.7) | | 3.3 | |
| Vitamin A (RAE)^2^, µg | 17.0 (7.2-30.4) | 18.7 (10.3-28.7) | | 9.5 | | 46.4 (30.9-59.0) | | | 46.6 (33.1-60.2) | | 0.3 | |
| ^1^ In Nepal, the recall aid was consulted directly after the first pass of the 24HR. In Senegal, the recall aid was consulted after all four passes of the 24HR. Values in Columns A and B are median (inter-quartile range). Values in Columns C are percent difference of medians, calculated as: B-A/A*100. Bivariate, cluster-controlled regressions of log-transformed data are used to compare caregivers in Column A with those Column B (unpaired data), controlling for variables with p<0.05 in Table 1 (Nepal: caregiver age, no formal education, paid work in the last month; Senegal: child spent time away from caregiver yesterday; household is food secure). Significant differences between the two groups concerned are shown in Columns C when *** p<0.001,** p<0.01, * p<0.05 for the recall aid use coefficient. | | | | | | | | | | | | |
| ^2^ RAE = retinol activity equivalents.  ^3^ When adding in controls for interviewer during sensitivity analyses, effect size becomes statistically significant at p<0.01 rather than p<0.05 level. | | | | | | | | | | | | |
