# Supplementary figures and images for: The Use of Pictorial Recall Aids Modifies Dietary Assessment Results: Experiences from Quantitative 24-hour Dietary Recalls of Young Children in Nepal and Senegal
Source: Curr Dev Nutr. 2024 Aug 30;9(Suppl 1):104452. doi: 10.1016/j.cdnut.2024.104452 (PMC12125692; doi:10.1016/j.cdnut.2024.104452)

**Supplementary Figure 1: Nepal recall aid (English version)**

**
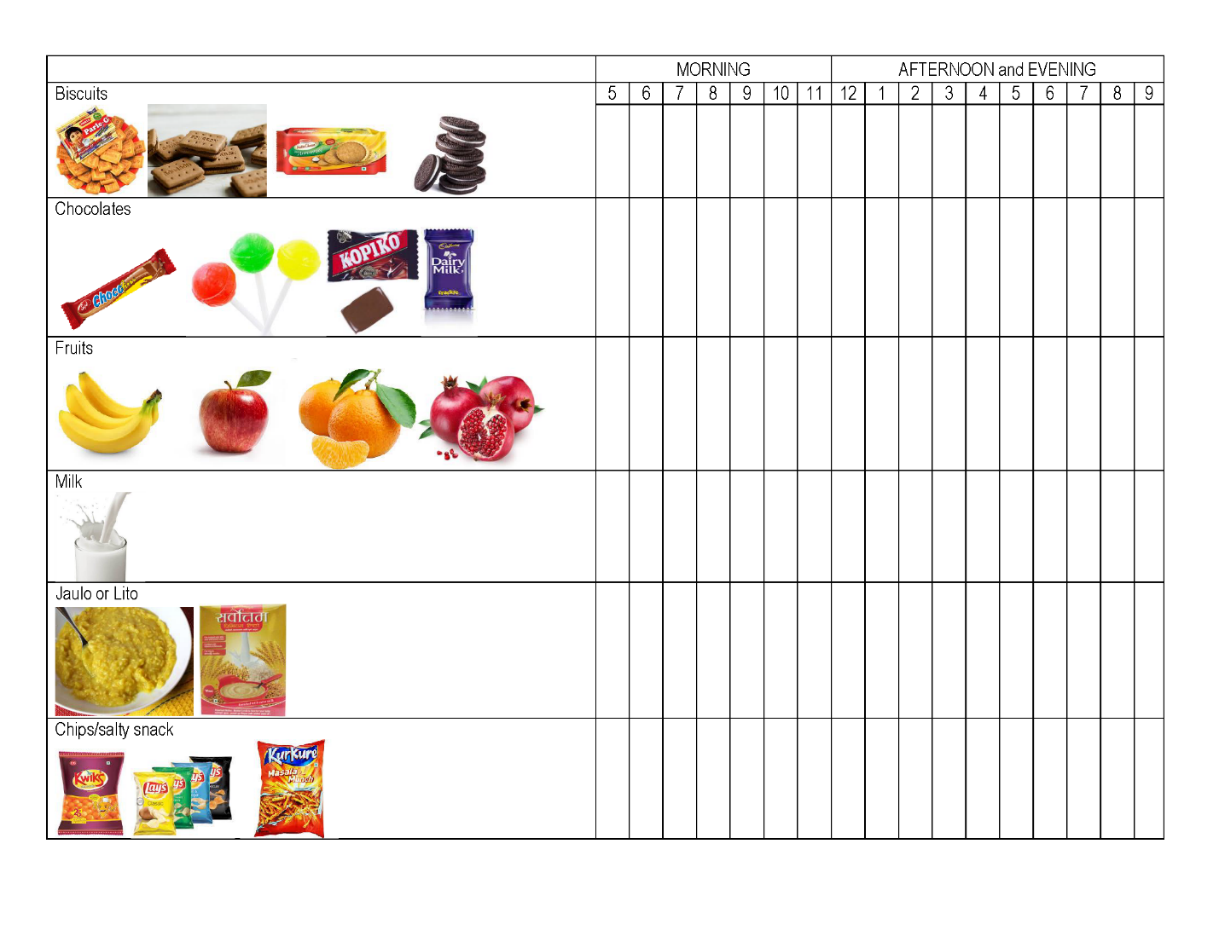
**

Supplement: multimedia component 2 [file mmc2.docx]

**Supplementary Figure 2: Senegal recall aid (English version)**


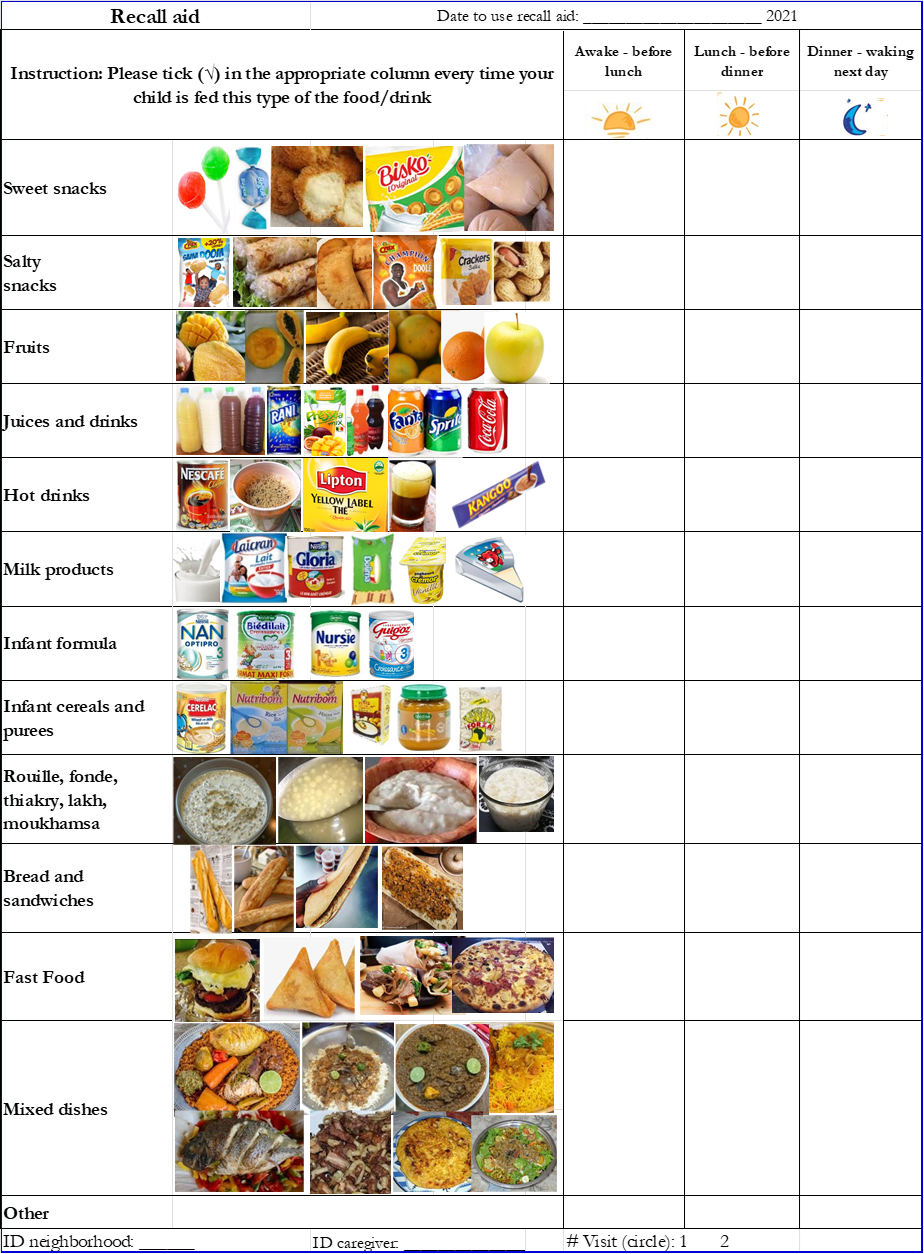

Supplement: multimedia component 3 [file mmc3.docx]
